# Black and Yellow Soybean Consumption Prevents High-Fat Diet-Induced Obesity by Regulating Lipid Metabolism in C57BL/6 Mice

**DOI:** 10.1155/2023/6139667

**Published:** 2023-04-18

**Authors:** Eun Woo Jeong, Sanjeev Kumar Dhungana, Yun Sun Yang, Youjin Baek, Jeong-Hyun Seo, Beom-Kyu Kang, Chan-Sik Jung, Sang-Ik Han, Hyeon Gyu Lee

**Affiliations:** ^1^Department of Food and Nutrition, Hanyang University, Seoul 04763, Republic of Korea; ^2^Department of Southern Area Crop Science, National Institute of Crop Science, Rural Development Administration, Miryang 50424, Republic of Korea

## Abstract

To evaluate the antiobesity effects of yellow and black soybean, C57BL/6 mice were provided with a normal diet, high-fat diet, HFD-containing yellow soybean powder (YS), and black soybean powder (BS) for six weeks. Compared with the HFD group, both YS and BS decreased body weight by 30.1% and 37.2% and fat in tissue by 33.3% and 55.8%, respectively. Simultaneously, both soybeans significantly reduced the serum triglyceride and total cholesterol levels and regulated the lipogenic mRNA expressions of Ppar*γ*, Acc, and Fas genes in the liver, supporting reduced body adiposity. Furthermore, BS significantly increased Pgc-1*α* and Ucp1 mRNA expression levels in epididymal adipose tissue, indicating thermogenesis is the key mechanism of BS. Taken together, our findings suggest that both soybeans prevent high-fat diet-induced obesity in mice by regulating lipid metabolism, and BS, in particular, has a greater antiobesity potential than YS.

## 1. Introduction

Obesity, defined as a state of excessive fat accumulation, is a major public health issue with over 2.2 billion people worldwide being overweight or obese [1]. Obesity contributes to elevate healthcare costs, lost productivity, and adverse social and economic outcomes [2]. Moreover, many studies have confirmed that obesity is strongly associated with metabolic syndrome, including type 2 diabetes, dyslipidemia, hypertension, and cardiovascular disease [3–5]. Therefore, obesity threatens public health and contributes to a decrease in quality of life and an increase in mortality.

Currently, various medicines, such as orlistat, lorcaserin, and sibutramine, have been used to treat obesity by controlling appetite, lipid absorption, hormone action, and lipid metabolism [6]. Nevertheless, synthetic antiobesity drugs may have undesirable side effects, such as vomiting, insomnia, dry mouth, stomachache, and headache [7]. Thus, preventing and treating obesity is still challenging. It has been suggested that the most effective way to combat obesity is to make appropriate diet and lifestyle changes, but it is difficult to change long-term lifestyles. Thus, dietary intervention with functional foods for preventing or treating obesity has been highlighted [8, 9].

Soybean (Glycine max) has been widely consumed in Asian countries. Soybeans contain a high amount of protein, carbohydrates, dietary fiber, vitamins and minerals, and phytochemicals, including isoflavones (i.e., daidzein, genistein, and glycitein), saponins, phenolic acids, and anthocyanin [10]. Due to the various components of soybean, previous studies have reported its health-promoting effects such as anti-inflammatory, antihypertensive, and cardiovascular protective activities [11]. Moreover, prior studies have revealed the antiobesity effects of using soybean in different forms and its components. For instance, black soybean seed coat extract inhibited excessive fat accumulation in mice fed high-fat diet [12, 13]. In addition, soybean peptides ameliorated obesity in obese mice and rats via reduction of endoplasmic reticulum stress, upregulation of leptin-like signaling, or AMP-activated protein kinase activation [14, 15]. Moreover, anthocyanin in black soybean prevents obesity through modulating appetite regulatory mechanisms or alleviating oxidative stress and inflammation [16, 17]. Overall, the components of soybean have an ant-obesity activity; however, the effects of soybean, as a whole food, on high-fat diet-induced obesity is not yet clear and still needs to be figured out. Therefore, we aimed to evaluate the antiobesity effects of a soybean-enriched diet and the possible mechanism. We also compared the effects of yellow soybean (as a conventional soybean) and black soybean on high-fat diet-induced obesity.

## 2. Materials and Methods

### 2.1. Experimental Animals and Diets

Four-week-old male C57BL/6 mice were purchased from Koatech (Pyeongtaek, Korea). The mice were housed in a controlled condition (22 ± 1°C, 50 ± 10% relative humidity, 150–300 lux light, and 12 h day-night cycle). The mice were given free access to food and water. The animals were acclimatized for one week and divided into four groups (*n* = 10): normal diet (ND), high-fat diet (HFD), HFD mixed with yellow soybean powder (YS), and HFD mixed with black soybean powder (BS). Yellow soybean (Glycine max L., Daewon) and black soybean (Glycine max L., Cheongja#5) were obtained from the National Institute of Crop Science (Rural Development Administration, Miryang, Korea). The ND group was fed with a normal diet (Pico-Lab rodent diet 20 5053, Lab diet, St. Louis, MO, USA). Other groups were fed with a high-fat diet (D12492, 60% kcal from fat) (Research Diets, New Brunswick, NJ, USA) and its modification. For YS and BS groups, a high-fat diet was formulated with 50% (w/w) of each soybean powder and adjusted using the proximate composition of yellow and black soybean powder with protein (39.4% and 40.7%), carbohydrates (25.5% and 25.2%), and fat contents (18.9% and 17.8%), respectively. The mixture percentage of soybean was determined by the previous studies [18, 19]. All groups were supplemented with each diet for six weeks (Table S1). The nutritional composition of yellow and black soybean was provided by the National Institute of Crop Science in Rural Development Administration (Miryang, Korea) (Table S2). The animal experiments were approved by the Institutional Animal Care and Use Committee (IACUC) of Hanyang University (HY-IACUC-2020-0169A).

### 2.2. Growth Performance

The body weight and food intake were measured once and twice a week, respectively. The food efficiency ratio (FER) was calculated as the ratio of body weight gain to the total food intake (FER = body weight gain (g)/food intake (g) × 100).

### 2.3. Body Composition Analysis and Organ Weights

At the end of the experimental period, the mice were fasted for 12 h and anesthetized with a mixture of 10 mg/kg bw of xylazine (Bayer Korea, Seoul, Korea) and 100 mg/kg bw of ketamine (Yuhan Co., Seoul, Korea). The fat in tissue (%) and lean mass (%) were measured using dual-energy X-ray absorptiometry (DEXA, Medikors, Seongnam, Korea). The lean mass (%) was calculated as the ratio of lean mass to body weight. The liver and epididymal adipose tissue were immediately isolated, weighed, and stored at −80°C for further analysis.

### 2.4. Blood Biomarkers Analysis

Blood was collected from the retro-orbital plexus and immediately centrifuged at 3,000 × g for 15 min to obtain a serum. In addition, the liver function indicators such as aspartate transaminase (AST) and alanine transaminase (ALT) in the serum were measured using a commercial kit (Asan Pharmaceutical, Seoul, Korea) following the manufacturer's instructions. The serum triglyceride (TG) and total cholesterol (TC) were analyzed by the automatic blood biochemical analyzer (Fujifilm, Tokyo, Japan).

### 2.5. Quantitative Real-Time Polymerase Chain Reaction (qRT-PCR) Analysis

The total RNA from the liver and epididymal adipose tissue was obtained using Trizol (Ambion, Austin, TX, USA). The cDNA was synthesized with purified RNA using a Prime Script ™ RT reagent kit (Takara, Shiga, Japan). The PCR amplification was performed with 2 *μ*L of the sample mixed with cDNA, primers, and SYBR green using the CFX96TM RT-PCR detection system (Bio-Rad, Hercules, CA, USA). Each value was normalized to 36b4, and the differences in mRNA expression levels of the genes were calculated using the delta-delta threshold cycle method compared with the HFD group. The sequences of the primer used in this study are presented in Table 1.

### 2.6. Statistical Analysis

All data are presented as the mean ± standard deviation. The statistical comparisons among the groups were performed one-way ANOVA with Tukey's post-hoc tests using the Prism 9 program (GraphPad Software, La Jolla, CA, USA). *p* < 0.05 was considered to be statistically significant.

## 3. Results

### 3.1. Growth Performance

The growth performance of mice is presented in Table 2. The final body weight, weight gain, and food efficiency ratio in the HFD group extremely increased compared with those in the ND group (*p* < 0.05). Both soybean seeds dramatically decreased body weight, weight gain, and food efficiency ratio compared with the HFD group without alteration of food and energy intake (*p* < 0.05). Interestingly, the BS group showed lower body weight, weight gain, and feed efficiency ratio than those in the YS group (*p* < 0.05). Thus, the BS intake effectively prevented weight gain induced by a high-fat diet compared to YS.

### 3.2. Body Composition

**Figure 1:**
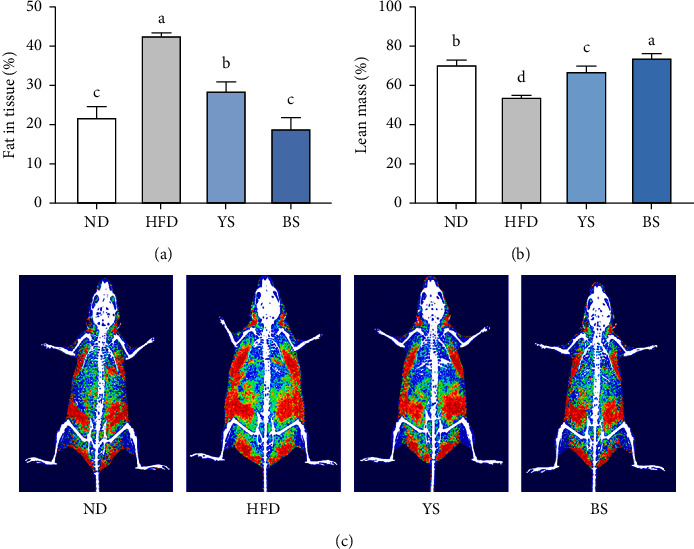
(a) Fat in tissue (%), (b) lean mass (%), and (c) body composition of C57BL/6 mice fed with a high-fat diet mixed with yellow or black soybean powders. Red and green areas represent fat content and lean body content, respectively. Data are expressed as mean ± standard deviation (*n* = 8). ^(a–d)^Different lowercase letters above the bars represent significant differences by Tukey's post-hoc test (*p* < 0.05). ND: normal diet (13% kcal from fat); HFD: high-fat diet (60% kcal from fat); YS: high-fat diet mixed with yellow soybean powder; BS: high-fat diet mixed with black soybean powder.

The DEXA analysis was performed to assess whether weight loss in soybean-fed groups was owing to fat loss (Figure 1). The YS and BS groups were found to remarkably reduce fat in tissue (%) compared with the HFD group (*p* < 0.05). Besides, lean mass (%) was significantly (*p* < 0.05) increased by 23.6% and 36.2% in YS and BS groups compared with the HFD group, respectively. The BS group, in particular, had significantly lower fat in tissue and higher lean mass than the YS group (*p* < 0.05).

### 3.3. Liver and Epididymal Adipose Tissue Weight

**Figure 2:**
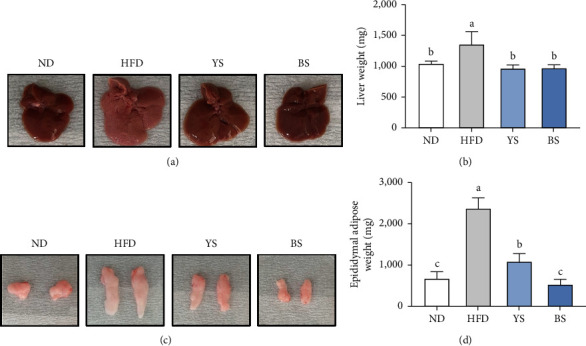
(a) Representative liver morphology photos, (b) liver weight, (c) representative epididymal adipose tissue morphology photos, and (d) epididymal adipose tissue weight of C57BL/6 mice fed with a high-fat diet mixed with yellow or black soybean powders. Data are expressed as mean ± standard deviation (*n* = 8). ^(a–c)^ Different lowercase letters above the bars represent significant differences by Tukey's post-hoc test (*p* < 0.05). ND: normal diet (13% kcal from fat); HFD: high-fat diet (60% kcal from fat); YS: high-fat diet mixed with yellow soybean powder; BS: high-fat diet mixed with black soybean powder.

The morphology and weights of liver and epididymal adipose tissue are shown in Figure 2. When the liver of the HFD group was observed immediately after the organ harvest, it was pale red compared to the other groups, and it had the fat dispersed, indicating a typical fatty liver; however, YS and BS reversed the liver color similar to the ND group. The liver and epididymal adipose tissue weights of the HFD group (1,355 mg and 2,367 mg) were significantly greater than those of the ND group (1,041 mg and 543 mg) (*p* < 0.05). The liver and epididymal adipose tissue weights of the YS (964.1 mg and 1,087 mg) and BS (970.2 mg and 543.1 mg) groups were remarkably lower compared with those of the HFD group (*p* < 0.05). Furthermore, the BS group showed significantly lower epididymal adipose tissue weight than the YS group, similar to the ND group (*p* < 0.05).

### 3.4. Blood Biomarker Profiles

The hepatotoxicity index and lipid profiles in the serum are shown in Table 3. The concentrations of aspartate transaminase (AST) and alanine transaminase (ALT) were notably increased in the HFD group compared with the ND group (*p* < 0.05). The AST level was significantly reduced in the BS group and the ALT level decreased in both soybean groups compared with the HFD group (*p* < 0.05). Also, the triglyceride (TG) and total cholesterol (TC) levels in the HFD group were higher than those in the ND group (*p* < 0.05). Both YS and BS groups showed a significant decrement in TG and TC levels compared with the HFD group (*p* < 0.05). Overall, there were no significant differences in the blood biomarker profiles between the YS and BS groups.

### 3.5. Gene Expression in Liver and Epididymal Adipose Tissues

**Figure 3:**
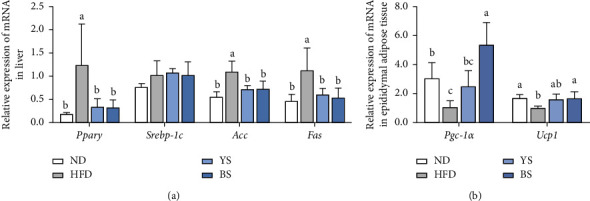
mRNA expression levels related to (a) lipogenesis in the liver, (b) thermogenesis in the epididymal adipose tissues of C57BL/6 mice fed with a high-fat diet mixed with yellow or black soybean powders. Data are expressed as mean ± standard deviation (*n* = 8). ^(a–c)^Different lowercase letters above the bars represent significant differences by Tukey's post-hoc test (*p* < 0.05). ND: normal diet (13% kcal from fat); HFD: high-fat diet (60% kcal from fat); YS: high-fat diet mixed with yellow soybean powder; BS: high-fat diet mixed with black soybean powder.

To explore the mechanism underlying the antiobesity effects of YS and BS, the mRNA expression level of hepatic lipogenesis associated genes in the liver (Figure 3(a)) and thermogenesis associated genes in epididymal adipose tissue (Figure 3(b)) were measured by the qRT-PCR method. We determined the mRNA expression levels of Ppar*γ*, Srebp-1c, Acc, and Fas, which are transcription factors and enzymes involved in de novo lipogenesis. Compared with the ND group, the HFD group significantly upregulated Ppar*γ*, Acc, and Fas (*p* < 0.05). The YS and BS groups showed remarkably decreased expression levels of *Pparγ*, *Acc*, and *Fas* compared with the HFD group (*p* < 0.05). Otherwise, Srebp-1c expression levels were not significantly different among all groups. Overall, there were no differences in mRNA expression levels of lipogenic genes between the YS and BS groups. Interestingly, Pgc-1*α* and Ucp1 mRNA expression levels related to thermogenesis were significantly (*p* < 0.05) increased in the BS group compared with those of the HFD group; however, Pgc-1*α* and Ucp1 mRNA expression levels were not significantly altered in the YS group.

## 4. Discussion

In this study, the authors assessed the effects of YS and BS on adiposity, biochemical parameters, and the regulation of hepatic and adipose tissue gene expressions in mice. The current study demonstrated that the YS and BS groups had reduced adiposity and improved blood biochemical parameters related to hepatotoxicity and lipid profiles. In total, these data demonstrated that both soybeans' consumption can prevent HFD-induced obesity in mice.

Interestingly, the BS group was more effective in preventing HFD-induced obesity than the YS group. BS significantly decreased body weight gain, FER, fat in tissue (%), and epididymal adipose tissue, and it increased lean mass (%) compared with YS. BS has been reported to have various health-promoting effects due to its high contents of polyphenols such as anthocyanin, soyasaponin, and isoflavones. According to previous studies, black soybean seed coat contains high contents of anthocyanin (cyanidin-3-glucoside, cyanidin-3-O-galactoside, and peonidin-3-O-glucoside) compared with yellow soybean [20, 21]. In addition, black soybean (Cheongja) had higher contents of soyasaponins and isoflavones than yellow soybean (Daewon), which are the same cultivars used in this study [22]. Previous studies have shown that anthocyanin inhibited fat accumulation in mice fed HFD [13, 17]. Cyanidin-3-glucoside, the major anthocyanin in black soybean, decreased body weight and white adipose tissue weight in db/db mice [23]. Besides, soyasaponin significantly decreased body weight by 7% and relative adipose tissue weight by 35% with suppressing lipogenesis in epididymal adipose tissue of obese mice [24]. The high content of isoflavones with soy protein diet significantly reduced body weight and total body fat compared with low content of isoflavones with soy protein diet in the obese ZDF rats [25]. In particular, daidzein, one of the main soy isoflavones, significantly lowered weight gain with high leptin and low adiponectin levels in HFD-induced obese rats [26]. Taken together, our results suggest that BS exhibits more powerful antiobesity effects due to different phytochemical profiles compared with YS.

Obesity is associated with lipid metabolism abnormality since fatty acids from high-fat intake enter the liver to synthesize TG, which can lead to fatty liver and hyperlipidemia. Consumption of large quantities of HFD results in fat accumulation in the liver, thereby damaging it and potentially exposing it to nonalcoholic fatty liver disease [3, 27]. Hepatic lipid accumulation can be increased by activation of transcription factors such as Ppar*γ* and Srebp-1c [28, 29]. Ppar*γ* induces the expression of genes such as Fabp4, which controls fatty acid uptake and TG synthesis in the liver. Srebp-1c also regulates the genes such as Acc and Fas that control fatty acid and TG synthesis. The current study shows that supplementation with both soybeans significantly decreased the liver weight and serum ALT, TG, and TC levels. Moreover, both soybeans suppressed the mRNA expression levels of Ppar*γ*, Acc, and Fas, supporting reduced body adiposity. These results are associated with a previous study reporting that dietary soy protein is effective in reducing the activities of hepatic lipogenic enzymes and inhibiting the hepatic fatty acid synthesis in rats [30]. In particular, soy *β*-conglycinin attenuates fatty liver and hyperlipidemia caused by a high-fat or high-cholesterol diet in mice and rats [31–33]. Soy *β*-conglycinin also lowered liver weight and serum TG through a decrease of fatty acid synthase activity and an augment of fecal TG excretion in KKAy mice [34]. However, the mRNA expression of Srebp-1c was not affected by the supplement of both soybeans. It may be due to the fact that Srebp-1c is a gene whose activity is modulated by proteolytic cleavage and posttranslational modification, thus mRNA expression of Srebp-1c may not always support the activity of Srebp-1c, as previously reported [35–37]. Taken together, both soybeans significantly decreased liver weight and serum lipid profiles. Furthermore, the down-regulation of lipogenic mRNA expression could be attributed to the reduction in adiposity in YS and BS groups. These results suggest that both soybeans may have a beneficial effect on obesity by improving serum lipid profiles and modulating lipid metabolism.

Recently, the browning of the white adipose tissue has been suggested to be promising strategy for preventing or treating obesity in that it increases energy expenditure by heat production [38]. Ucp1 is a transporter in the inner membrane of mitochondria that uncouples electron transport from ATP production, resulting in producing energy as heat [39]. The increased expression of Ucp1 in white adipose tissue could be involved in the browning process [40, 41]. Besides, Pgc-1*α*, a coactivator of Ppar*γ*, has been identified that stimulates mitochondrial biogenesis and cellular respiration by elevating the expression of Ucp1 [42]. The HFD downregulates Pgc-1*α* and Ucp1 mRNA expression in adipose tissue, resulting in decreased energy expenditure and increased diet-induced obesity. Notably, we found that BS significantly up-regulated Pgc-1*α* and Ucp1 mRNA expression level, indicating thermogenesis is one of the key anti-obesity mechanism of BS. These findings were consistent with the previous reports that black soybean seed coats up-regulated Pgc-1*α* and Ucp1 in adipose tissue [12, 43]. Additionally, black soybean seed coat increased Ucp2 protein expression level compared with yellow soybean seed coat in peritesticular fat of HFD-induced mice, although Ucp2 expression was not measured in this study [44]. Taken together, these results can provide convincing evidence that BS increased the mRNA expression related to thermogenesis in adipose tissue, which may be a reason for its stronger antiobesity effects compared to the YS group.

## 5. Conclusions

In conclusion, our study demonstrated that both soybeans prevented HFD-induced obesity and improved its related parameters in mice, but overall antiobesity effects of BS were stronger than YS. Mechanism studies have shown that both soybeans decreased lipogenic mRNA expression, supporting reduced body adiposity. In addition, BS promoted the mRNA expressions related to thermogenesis in epididymal adipose tissue, indicating that thermogenesis is the one of the key mechanism of BS. Taken together, these results suggest that both soybeans prevent HFD-induced obesity and BS exerts powerful antiobesity effects compared with YS. Furthermore, this study could be important to greatly expand our understanding of the nutraceutical potential of soybean.

## Figures and Tables

**Table 1:** qRT-PCR primer sequences used in mRNA expression analysis.

Gene	Forward (5′-3′)	Reverse (5′-3′)
Ppar*γ*	CGCTGATGCACTGCCTATGA	AGAGGTCCACAGAGCTGATTCC
Srebp-1c	GAACAGACACTGGCCGAGAT	GAGGCCAGAGAAGCAGAAGAG
Acc	GCCTCTTCCTGACAAACGAG	TAAGGACTGTGCCTGGAACC
Fas	AGCACTGCCTTCGGTTCAGTC	AAGAGCTGTGGAGGCCACTTG
Pgc-1*α*	TATGGAGTGACATAGAGTGTGCT	CCACTTCAATCCACCCAGAAAG
Ucp1	GGCAAAAACAGAAGGATTGC	TAAGCCGGCTGAGATCTTGT
36b4	TCTAGGACCCGAGAAGACCTC	GTTGTCAAACACCTGCTGGAT

**Table 2:** Growth performance of C57BL/6 mice fed with a high-fat diet mixed with yellow or black soybean powder.

Group	ND	HFD	YS	BS
Initial body weight (g)	20.98 ± 0.59	20.63 ± 0.78	20.55 ± 0.79	20.63 ± 0.81
Final body weight (g)	26.61 ± 1.88^bc^	39.6 ± 1.10^a^	27.7 ± 0.45^b^	24.9 ± 1.67^c^
Body weight gain (g)	5.89 ± 1.11^bc^	18.80 ± 0.67^a^	6.92 ± 1.35^b^	4.23 ± 2.19^c^
Feed intake (g/day)	3.18 ± 0.27	2.84 ± 0.04	2.55 ± 0.32	2.80 ± 0.22
Energy intake (kcal/day)	10.84 ± 0.24	13.79 ± 0.17	13.11 ± 1.64	14.39 ± 0.13
Feed efficiency ratio	4.41 ± 0.82^c^	15.73 ± 0.60^a^	6.68 ± 1.05^b^	3.59 ± 1.80^c^

Data are expressed as mean ± standard deviation (*n* = 8). ^a–c^The values with different lowercase letters in the same column represent significant differences by Tukey's post-hoc test (*p* < 0.05). ND: normal diet (13% kcal from fat); HFD: high-fat diet (60% kcal from fat); YS: high-fat diet mixed with yellow soybean powder; BS: high-fat diet mixed with black soybean powder.

**Table 3:** Blood biomarker profiles in C57BL/6 mice fed with a high-fat diet mixed with yellow or black soybean powders.

Group	ND	HFD	YS	BS
AST (IU/L)	6.60 ± 1.78^b^	8.72 ± 1.11^a^	7.38 ± 2.29^ab^	6.54 ± 0.81^b^
ALT (IU/L)	11.70 ± 3.21^b^	15.48 ± 2.91^a^	8.79 ± 1.34^b^	8.64 ± 2.22^b^
TG (mg/dL)	84.0 ± 5.03^c^	178.9 ± 2.85^a^	113.8 ± 9.31^b^	122.4 ± 13.46^b^
TC (mg/dL)	76.8 ± 4.89^c^	163.3 ± 6.56^a^	99.8 ± 6.43^b^	106.8 ± 9.00^b^

Data are expressed as mean ± standard deviation (*n* = 8). ^(a–c)^The values with different lowercase letters in the same column represent significant differences by Tukey's post-hoc test (*p* < 0.05). ND: normal diet (13% kcal from fat); HFD: high-fat diet (60% kcal from fat); YS: high-fat diet mixed with yellow soybean powder; BS: high-fat diet mixed with black soybean powder.

## Data Availability

The data that support the findings of this study are available on request from the corresponding author.

## Abbreviations

Acc: Acetyl-CoA carboxylase
Fabp4: Fatty acid-binding protein 4
Fas: Fatty acid synthase
Pgc-1*α*: Peroxisome proliferator-activated receptor gamma coactivator 1 alpha
Ppar*γ*: Peroxisome proliferator-activated receptor gamma
Srebp-1c: Sterol regulatory element binding protein-1c
Ucp: Uncoupling protein.

## Supplementary Materials

Supplementary Materials Table S1. Composition of experimental diets (g/100 g diet); Table S2. Chemical composition of yellow and black soybeans.
