# Supplementary material for: Black and Yellow Soybean Consumption Prevents High-Fat Diet-Induced Obesity by Regulating Lipid Metabolism in C57BL/6 Mice
Source: Evid Based Complement Alternat Med. 2023 Apr 18;2023:6139667. doi: 10.1155/2023/6139667 (PMC10129420; doi:10.1155/2023/6139667)
Supplement: Supplementary Materials — Table S1. Composition of experimental diets (g/100 g diet); Table S2. Chemical composition of yellow and black soybeans. [file 6139667.f1.docx]

Table S1. Composition of experimental diets (g/100 g diet)

| Component | ND | HFD | YS | BS |
| --- | --- | --- | --- | --- |
| Casein | - | 24 | 5.7 | 5.1 |
| L-cystine | - | 0.4 | 0.4 | 0.4 |
| Corn starch | - | 0 | 0 | 0 |
| Maltodextrin 10 | - | 15 | 3.1 | 3.3 |
| Sucrose | - | 8.3 | 8.7 | 8.7 |
| Cellulose | - | 13.2 | 0 | 0 |
| Soybean oil | - | 3 | 3.2 | 3.2 |
| Lard | - | 29.4 | 21.7 | 22.2 |
| Mineral mix S10026 | - | 1.2 | 1.3 | 1.3 |
| Dicalcium phosphate | - | 1.6 | 1.7 | 1.6 |
| Calcium carbonate | - | 0.7 | 0.7 | 0.7 |
| Potassium citrate, 1H2O | - | 2 | 2.1 | 2.1 |
| Vitamin mix V 10001 | - | 1.2 | 1.3 | 1.3 |
| Choline bitartrate | - | 0.2 | 0.3 | 0.3 |
| Yellow soybean powder | - | 0 | 50 | 0 |
| Black soybean powder | - | 0 | 0 | 50 |
| Protein (kcal, %) | 25 | 20 | 20 | 20 |
| Carbohydrate (kcal, %) | 62 | 20 | 20 | 20 |
| Fat (kcal, %) | 13 | 60 | 60 | 60 |
| Total | 100 | 100 | 100 | 100 |

ND: normal diet (13% kcal from fat); HFD: high-fat diet (60% kcal from fat); YS: high-fat diet mixed with yellow soybean powder; BS: high-fat diet mixed with black soybean powder. Composition of normal diet is not provided from the supplier.

Table S2. Chemical composition of yellow and black soybeans

| Component | YS | BS |
| --- | --- | --- |
| Fatty acid (% crude fat) | | |
| Palmitic acid (16:0) | 11.18 ± 0.03 ^b^ | 11.83 ± 0.03 ^a^ |
| Stearic acid (18:0) | 4.10 ± 0.05 ^b^ | 3.38 ± 0.02 ^a^ |
| Oleic acid (18:1) | 19.74 ± 0.21 ^b^ | 16.53 ± 0.09 ^a^ |
| Linoleic acid (18:2) | 56.99 ± 0.31 | 58.59 ± 0.10 |
| Linolenic acid (18:3) | 7.99 ± 0.08 ^b^ | 9.66 ± 0.04 ^a^ |
| Sugar (mg/g dry basis) | | |
| Fructose | 0.65 ± 0.01 ^b^ | 1.55 ± 0.04 ^a^ |
| Galactose | 2.07 ± 0.13 | 2.21 ± 0.41 |
| Glucose | 3.52 ± 0.23 ^b^ | 4.15 ± 0.32 ^a^ |
| Raffinose | 6.63 ± 0.68 | 6.72 ± 0.24 |
| Stachyose | 22.14 ± 0.75 ^a^ | 20.52 ± 0.42 ^b^ |
| Sucrose | 28.81 ± 3.02 ^b^ | 45.22 ± 3.34 ^a^ |
| Amino acid (mg/100 g dry basis) | | |
| Alanine | 16.59 ± 0.15 ^a^ | 14.22 ± 0.17 ^b^ |
| Asparagine | 23.46 ± 0.01 ^a^ | 19.89 ± 0.15 ^b^ |
| Arginine | 28.70 ± 0.12 ^a^ | 26.95 ± 0.17 ^b^ |
| Aspartic acid | 43.45 ± 0.62 ^a^ | 38.70 ± 0.27 ^b^ |
| Cysteine | 3.36 ± 0.25 | 1.90 ± 0.02 |
| Glutamic acid | 76.20 ± 1.16 ^a^ | 67.50 ± 0.47 ^b^ |
| Glycine | 16.57 ± 0.05 ^a^ | 14.09 ± 0.22 ^b^ |
| Histidine | 10.05 ± 0.00 ^a^ | 8.90 ± 0.04 ^b^ |
| Isoleucine | 16.86 ± 0.12 ^a^ | 14.94 ± 0.06 ^b^ |
| Leucine | 30.08 ± 0.13 ^a^ | 26.40 ± 0.18 ^b^ |
| Lysine | 24.31 ± 0.03 ^a^ | 21.75 ± 0.16 ^b^ |
| Methionine | 4.30 ± 0.03 ^a^ | 3.91 ± 0.04 ^b^ |
| Phenylalanine | 19.79 ± 0.11 ^a^ | 17.05 ± 0.09 ^b^ |
| Proline | 18.44 ± 0.38 ^a^ | 16.58 ± 0.23 ^b^ |
| Serine | 20.22 ± 0.28 ^a^ | 18.03 ± 0.14 ^b^ |
| Threonine | 14.79 ± 0.20 ^a^ | 12.75 ± 0.11 ^b^ |
| Tyrosine | 12.27 ± 0.02 ^a^ | 10.45 ± 0.02 ^b^ |
| Valine | 17.77 ± 0.11 ^a^ | 15.80 ± 0.23 ^b^ |
| Isoflavone (μg/g dry basis) | | |
| Daidzein | 36.42 ± 5.31 ^b^ | 192.20 ± 53.05 ^a^ |
| Glycitin | 32.76 ± 4.86 | 34.90 ± 3.40 |
| Genistein | 78.80 ± 10.64 ^b^ | 207.56 ± 59.23 ^a^ |
| Malonyldaidzin | 249.23 ± 60.97 ^b^ | 1282.45 ± 78.95 ^a^ |
| Malonylglycitin | 54.74 ± 11.71 | 59.17 ± 6.70 |
| Malonylgenistin | 469.02 ± 82.29 ^b^ | 1138.28 ± 70.38 ^a^ |
| Genistin | 31.17 ± 9.26 ^b^ | 74.90 ± 1.7.09 ^a^ |
| Glycitein | 18.85 ± 8.07 | 12.33 ± 2.92 |
| Anthocyanin (μg/g dry basis of seed coat) | | |
| Delphinidin-3-glucoside | 1427.52 ± 488.93 | ND |
| Cyanidin-3-O-galactoside | 132.51 ± 8.52 ^b^ | 258.23 ± 93.68 ^a^ |
| Cyanidin-3-glucoside | 3063.08 ± 535.23 ^b^ | 16483.43 ± 3159.25 ^a^ |
| Petunidin-3-O-glucoside | 1135.95 ± 279.02 ^a^ | 182.21 ± 6.59 ^b^ |
| Pelargonidin-3-glucoside | 164.25 ± 19.22 | 213.46 ± 14.02 |
| Peonidin-3-glucoside | 384.53 ± 31.42 ^b^ | 647.48 ± 62.57 ^a^ |

ND: not detected. YS: yellow soybean powder; BS: black soybean powder. Data are expressed as mean ± standard deviation. ^a-b)^ The values with different lowercase letters in the same row represent significant differences by Student’s t-test (*p*<0.05).

The chemical composition of yellow and black soybeans is provided by National Institute of Crop Science in Rural Development Administration (Miryang, Korea).
